# Legal challenges for the implementation of advanced clinical digital decision support systems in Europe

**Published:** 2018-08-18

**Authors:** Colin Mitchell, Corrette Ploem

**Affiliations:** ^1^*Academic Medical Center, University of Amsterdam, the Netherlands*; ^2^*Department of Public Health, Academic Medical Center, University of Amsterdam, the Netherlands*

**Keywords:** decision support systems, personalized medicine, data protection, privacy, medical law

## Abstract

Systems based on artificial intelligence and machine learning that facilitate decision making in health care are promising new tools in the era of ‘personalized’ or ‘precision’ medicine. As the volume of patient data and scientific evidence grows, these computerised decision support systems (DSS) have great potential to help healthcare professionals improve diagnosis and care for individual patients. However, the implementation of these tools in clinical care raises some foreseeable legal challenges for healthcare providers and DSS-suppliers in Europe: How does the use of complex and novel DSS relate to professional standards to provide a reasonable standard of care? What should be done in terms of testing before DSS can be used in regular practice? What are the potential liabilities of health care providers and DSS companies if a DSS fails to function well? How do legal requirements for the protection of patient data and general privacy rights apply to likely DSS scenarios? In this article, we provide an overview of the current law and its general implications for the use of DSS, from a European perspective. We conclude that healthcare providers and DSS-suppliers will have the best chance of meeting legal challenges if: they are first tested in translational research with the patients’ explicit, informed consent; DSS-suppliers and healthcare providers are able to clarify and agree on their individual legal responsibilities, and; patients are properly informed about privacy risks and able to decide themselves whether their data can be used for other purposes, or are stored and processed outside the EU. DSS developers and healthcare providers will need to work together closely to ensure compliance with national and European regulations and standards required for reasonable and safe patient care.

**Relevance for patients:**

Advanced digital decision support systems have the potential to improve patient diagnosis and care. In this article we discuss key legal issues to support translational research using DSS and ensure that they meet the high standards for protection of patient safety and privacy in Europe.

## 1. Introduction

The introduction of new technologies in the field of ge-nomics and genetics, such as next generation sequencing, and advances in medical care, screening and research are generat-ing more and more patient data. All this data has the potential to be very useful in developing the best possible care for pa-tients (‘personalized’ or ‘precision medicine’). However, most physicians and their teams lack the necessary time to consume all this information. This has been recognised by industry, which is now developing tools to help physicians quickly identifying the patient’s most relevant medical data, apply the latest medi-cal insights and explore the best treatment options. These tools are part of the broad category of clinical decision support sys-tems (DSS), defined by Berner and La Lande as ‘computer sys-tems designed to impact clinician decision making about indi-vidual patients at the point in time that these decisions are made [1].’ Such systems have been in existence for decades and have a wide range of functions, including (amongst others) provid-ing alerts or reminders, identifying drug-drug interactions, high-lighting specific guidelines at the point of care, and providing suggested courses of action to clinicians [2]. More advanced forms of these tools are increasingly driven by sophisticated ar-tificial intelligence (AI) or machine learning (ML). Some ex-amples are Google’s DeepMind [3], IBM’s Watson [4] and the Dutch system Oncoguide [5]. In this paper we focus especially on these advanced forms of DSS; systems which utilize artifi-cial intelligence and machine learning based on retrospective di-agnostic and therapeutic data from real patients. These new sys-tems raise specific challenges, for which the current legal frame-work may not be adequately prepared.

As with all new technologies in medicine, DSS create a ten-sion between the potential benefits, such as improved individual treatment, and the potential risks involved. Risks include errors in the use (or misuse) of the system, errors of analysis within the system, failures to secure confidentiality of information, or, breaches of patient privacy. In the case of genetic information, not only patients but also their relatives could be harmed. The overarching danger is of a loss of patient trust in the health care system if DSS errors occur. There are also wider concerns about the application of these tools that require careful consideration. For example, the users of DSS could regard the result from the computerised system as somehow ‘more valid, accurate or reli-able than human output’ [6]. This could intervene with a pro-cess of shared decision-making, and carefully weighing and dis-cussing test results and medical decisions in multidisciplinary teams or with other specialists in the field. Moreover, it is cer-tainly not unimaginable that patients or insurance companies de-mand the use of DSS to challenge clinical decisions, particularly if a DSS is perceived to favour or restrict treatment by compar-ison with the opinion of the treating physician. Such a devel-opment could put pressure on the fiduciary relationship between healthcare professionals and their patients.

We would like to stress that such risks are not new to medicine: digital decision support tools of various sorts have used for many years (even Medline and similar healthcare liter-ature databases are digital tools that are used for clinical decision making). However, the legal environment for medical databases is relatively simple, compared to that of providing medical care to patients; Whereas software and computer systems are usually regulated as products or devices in a limited manner [7], clinical DSS are situated in a much more rigorous regulatory context [8]. This means that the application of advanced DSS in healthcare will require compliance with a complex web of existing laws and regulations that will vary between countries, jurisdictions and re-gions (such as the European Union). The aim of this article is to identify and discuss some of the key legal issues related to the use of artificial-intelligence based DSS in Europe, to prevent le-gal problems occurring when these systems are implemented in clinical practice.

## 2. An overview of potential legal issues

### 2.1 Compliance with standard of care

A first key challenge for the implementation of DSS is how this will comply with the professional standards that apply to the care providers who will use them. In most legal systems, the re-quired standard is that of a reasonable and careful professional or healthcare provider. For example, according to Dutch law, when providing medical care, the doctor should act in confor-mity with the standards of a ‘good health care provider’, which means that he must observe the responsibilities laid upon him by the standards for (his category of) medical professionals [9]. The professional standard consists of relevant legislation, codes of conduct and other forms of self-regulation, possibly explained by jurisprudence. Similarly, in common law countries, such as the UK, the standard required of medical professionals is that of a reasonable degree of skill and care according to the standards of the profession [10]. On a European level, the explanatory report of the Council of Europe’s Convention for the Protection of Hu-man Rights and Dignity of the Human Being with regard to the Application of Biology and Medicine (hereafter: Biomedicine Convention) [11] of 1997 mentions that the standard of care may vary slightly from one society to another; ‘However, the funda-mental principles of the practice of medicine apply in all coun-tries. Doctors and, in general, all professionals who participate in a medical act (…) (…) must act with care and competence, and pay careful attention to the needs of each patient.’ In all ju-risdictions, guidelines, practice directions and practical guidance issued by professional bodies are very important in establishing a reasonable standard of care.

In the case of advanced DSS, which are – at least in sev-eral European countries – not yet even in their ‘research phase’, specific codes of conduct for practitioners are yet to be devel-oped. Translational research on the efficacy, safety and risks of using DSS is necessary (although not sufficient in itself) to es-tablish professional norms. One of the major questions of such research should be whether, and,in what way, DSS should be used in the different sectors of medical care? Perhaps the most likely, and moderate approach would be to use DSS as a ‘second opinion’ tool to facilitate medical teams that need to take very complex decisions about patients. For the complex decisions in-volved in personalized medicine, the outcome of DSS could be a welcome extra piece of information. A more extreme approach would be to use DSS as a decisive tool for diagnosing diseases and developing treatments for individual patients that largely re-place the individual decisions of the treating physician. Such use of DSS would challenge existing legal norms, for example that the main treating physician carries a final responsibility for medical decisions towards his/her patients, and rules about au-tomated decision-making under the EU General Data Protection Regulation (GDPR) [12]. Article 22 of the GDPR provides in-dividuals with the right not to be the subject of a decision based solely on automated processing which significantly affects him or her. This has the effect of a general prohibition on fully au-tomated decision-making (with no human involvement) [13], al-though there is room in the regulation for lawful automated deci-sion making, provided there has been explicit consent and there are suitable safeguards in place to allow the individual to express an opinion, contest a decision or obtain human intervention [14]. Translational research using DSS will need to meet ethical and legal requirements applying to research, as incorporated in the Declaration of Helsinki, and the European and national laws on human subject research. Important elements in this respect are written informed consent of research subjects, an approval of the research protocol from a review board or ethics committee and clear policies about what is done with the results of DSS. This raises some questions that require careful consideration, such as: what will be done with the research results? Will they be dis-cussed with the research participants? Are participants put at risk in a trial of DSS? How will privacy be secured and personal data protected?

### 2.2 Malfunctioning of the system

As Belard and colleagues identified, developers have been predominantly concerned with the efficacy of systems rather than with their safety, however, the application of health in-formation technology has not been without malfunctions with the potential for human harm [15]. Monitoring by the United States Food and Drug Administration (which regulates medi-cal devices) revealed four major categories of adverse events; ‘errors of commission, defined as accessing the wrong patient record or overwriting information (…) errors of omission, de-fined as loss or corruption of vital patient data (…) errors in the data analysis (…) incompatibility between multi-vendor appli-cation and systems…’[16] Some of these errors may appear ba-sic, but they are no less relevant in relation to DSS. There is the potential for inputting inadequate or incomplete patient infor-mation in the system, a flawed analysis by software, or, failures based on incompatibility with patient record systems. In par-ticular, it will be very difficult to determine whether algorithms used for the functioning of a DSS meet with the applicable pro-fessional standard of care within the country in which the sys-tem is used. Determining this will require that either the care providers are highly knowledgeable and able to critique the algo-rithms themselves, or, that the developer and/or supplying com-pany of a DSS (hereafter we use the term ‘DSS supplier’) can prove that the system is adequate in light of current professional standards. In other words: who carries (the main) responsibil-ity for problems caused by DSS in the course of regular medical treatment? It would make sense that the DSS supplier should be held responsible for technical and safety defects as they are the designers and developers of these tools [17].

In many countries, there has been significant debate whether software should be treated as a service or a product in law.[18] Now, the EU Medical Devices Regulation of 2017 [19] (which came into force 26th May 2017) [20] makes clear that software intended to provide information which is used to take decisions with diagnosis or therapeutic purposes, is classified as a medi-cal device [21]. DSS manufacturers will need to comply with many requirements, including that they have measures in place to provide financial compensation for potential liability for de-fective devices [22]. The regulation provides for a proportion-ate scrutiny of safety and efficacy for devices depending on their purpose and potential consequences. The highest levels of scrutiny applying to devices which provide information that is used to take decisions which could cause a serious deterio-ration in health, or even death [23]. For such DSS, safety will be assessed with a clinical evaluation prior to their entry into use and there is scope for clinical investigations of the device— essentially a form of research-to establish their safety and ef-ficacy [24]. This should reduce the potential for errors in the use of DSS and places clear responsibility on manufacturers to ensure safety and efficacy, but it cannot prevent all potential er-rors in the future, in particular errors in misuse of DSS by HCPs or misinterpretation of results. Pre-emptive clinical assessment also does not account for any changes that may occur in the al-gorithms and the potential for errors based on that.

A few final words about product liability. If we see DSS as a product, according to some legal systems (for example, UK law) liability will not require negligence but is instead based on ‘strict’ liability for defects. The DSS supplier may be liable even if they took reasonable care to avoid a defect or if they complied with professional standards [25]. This means a patient could re-cover damages if a DSS mistakenly suggested a course of course of action that lead to avoidable harm. The role of health care providers in this context is not likely to alter legal liability of DSS-suppliers because it would be foreseeable that health care providers would rely on the results of the analysis [26]. Inap-propriate use of DSS (or misinterpretation of results) would be a different matter, for which healthcare providers could be re-sponsible and liable too. Because the answers to these liability issues are complex and differ from jurisdiction to jurisdiction, we leave this issue to be further explored. We want to stress that it should be clear among all parties involved in DSS, where the responsibility of DSS-suppliers ends and where the responsibil-ity of health care provider begins.

### 2.3 Privacy, medical confidentiality and data protection

DSS process individual patient information from sources such as electronic health records in order to generate individ-ual diagnoses and results. One scenario is that this informa-tion is solely used for the patient’s diagnosis or treatment and this implies that it will not be shared with others than the team of healthcare providers treating the patient. However, in some cases the DSS-suppliers might retain this information and use it for further purposes, for example to improve their system, to conduct research (in cooperation with health care institutions) or even for commercial purposes. The latter use is, at least in many parts of Europe, a controversial issue and some hospitals will not be prepared to cooperate with companies for which commercial use is non-negotiable. With regard to privacy, it is essential that DSS-suppliers ensure the same level of confidentiality and pri-vacy protection as health care providers. All involved parties (not only the DSS-suppliers, but also all healthcare providers) should respect the rules of medical confidentiality and the prin-ciples governing the processing of health data [27]. A problem could be, however, that DSS-suppliers cannot ensure the same level of medical confidentiality as health care providers, for in-stance because patient data are held by them for purposes other than medical care, such as further improvement of their systems. In the latter situation, it is unclear whether (an employee of) a DSS-company could - on the basis of a physician’s right to tes-timonial privilege - refuse to provide access to the public au-thorities who request for such data. Another issue could be that data processing in relation to DSS will often be an international matter; involving cloud services, databases or data processing systems held in a different country to the country in which the DSS is used. This could include, for instance, that the data of pa-tients are stored in a database or cloud held within the territories of the United States and are thereby secured by less protective privacy regulations. If are transferred from an EU Member State to a third country, the provisions of chapter V of the GDPR of the European Union should be observed [28]. We will go into more detail on the implications of the GDPR below.

If data are genuinely anonymous they may be used with-out consent. However, if a person can be identified even in-directly, for example by combining several sources of informa-tion, the data should be treated as identifiable [29], and legal obligations, such as obtaining consent, apply. As stated above, ensuring compliance with medical confidentiality and other pri-vacy requirements becomes more challenging if data are used by DSS-providers for purposes other than patient care. If sensitive patient data are used within the healthcare institution to improve the operation of the system—which in turn should provide pa-tient benefit—, or is shared with DSS-suppliers for purely tech-nical operations with the data, this may be seen as analogous to auditing (monitoring and improvement) of the provided health care, and therefore the patient implicitly consented to this kind of use [30]. When data are used for purposes that go beyond quality assurance in a strict sense, such as scientific research, or when the DSS supplier use them for their own purposes, this may be an unlawful use of data without consent. A clear exam-ple of this is the case of the Royal Free London NHS Foundation Trust’s agreement with Google DeepMind. It demonstrates that there is a dividing line between reasonable use of patient data for improvement in care, and, a legally unreasonable, unexpected or disproportionate use. In this case, the Royal Free shared the identifiable data of 1.6 million patients without their consent, with DeepMind in order to test the safety of their Streams app, which was being developed to alert clinicians to patients at risk of acute kidney injury. The UK Information Commissioner ruled that this agreement failed to comply with data protection laws and that the Royal Free had failed to demonstrate a satisfactory legal basis for the processing of the sensitive personal data [31]. Taking each of the principles of data protection under the Euro-pean Data Protection Directive in turn [32], the Commissioner rejected that such a use of data could be lawfully based on the implied consent of 1.6 million patients and there was insufficient evidence to claim that the processing of data could be based on medical necessity. The Commissioner was not persuaded that it was necessary or proportionate to process this volume of data in order to test the clinical safety of the app. Furthermore, the Commissioner made clear that the lack of information and trans-parency towards patients would not allow them to prevent or opt-out of processing. Finally, the Royal Free had failed to im-plement a sufficiently detailed agreement with DeepMind to ensure that only the minimal possible data would be accessible to Deepmind and that processing would be conducted for limited means. Although the data protection principles in the GDPR remain largely the same as the principles in the former Data Pro-tection Directive, the new regulation is stricter on some points. For instance, it requires data controllers to carry out an impact assessment prior to processing—in particular, processing using new technologies—if it is likely to result in a high risk to the rights and freedoms of individuals [33]. In general, patient con-sent will be required in order to use identifiable patient informa-tion for purposes other than care, safeguarding quality and safety of care (in a strict sense), and management of health services [34]. We discuss this ‘secondary use’ of data used, and gener-ated, by DSS below. In some countries, such as the Netherlands, new laws on electronic data processing have been (or are being) introduced [35], requiring explicit consent the moment patient data are being exchanged between two or more individual health care institutions. The Dutch law does not apply to the exchange of patient data within one institution.

#### 2.3.1 Secondary uses of patient data

When special categories of personal data such as data concern-ing health, are processed on the basis of informed consent, the GDPR requires that the data subject has given explicit consent to the processing for one or more specified purposes [36]. The reg-ulation requires that consent is freely given, specific, informed and made with a clear and recorded affirmative act such as a writ-ten statement, electronic means or an oral statement [37]. The regulation specifically states that, when assessing whether con-sent is freely given, ‘utmost account shall be taken of whether, inter alia, the performance of a contract, including the provision of a service, is conditional on consent to the processing of per-sonal data that is not necessary for the performance of that con-tract [38].’ This is a warning that consent forms that require agreement to unrelated secondary processing of personal infor-mation in order to benefit from DSS could, quite easily, be seen as coercive and unlawful.

One of the exceptions to this rule is when medical data are to be processed for scientific research. In the Regulation’s pream-ble, the drafters of the Regulation make clear that scientific re-search should be facilitated by allowing processing of personal data for such purposes under certain conditions and safeguards set out in Union or Member State law [39]. This exception re-quires Member States to develop national law to implement it, with specific conditions. Article 9(2)(j) of the GDPR is more specific about these conditions: the law should be proportionate to the aim pursued; the essence of the right to data protection should be respected; and suitable and specific measures to safe-guard the fundamental rights and the interests of the data subject, such as pseudonymisation and data minimisation [40], should be provided. In the Netherlands, the Medical Treatment Agreement Act contains a few additional requirements, such as, if asking for consent is reasonably impossible or may not reasonably be required (because patients are deceased or untraceable, or the research involves enormous numbers of patients), then patients are offered an informed option to ‘opt-out’ from the use of their data. Additional requirements apply, such as that the research should serve a public interest.

#### 2.3.2 International transfer of patient data

A further challenge may occur where a DSS requires the trans-fer of patient data outside the EU, for example, to cloud storage in the USA or elsewhere. Current EU law and the GDPR pro-hibit the transfer of personal data outside the EU unless the Com-mission has decided there is an adequate level of protection, or, where there are adequate safeguards and effective legal remedies for the enforcement of data subjects rights.[41] Transfers might be adequately safeguarded using standard contractual clauses ap-proved by the Commission [42]. However, even a decision of adequacy by the Commission may be struck down, as occurred with the original EU-US Safe Harbor agreement which allowed US companies to self-certify that they would protect EU citizens data. A replacement, more robust, EU-US Privacy Shield agree-ment has been approved by the Commission but it is possible that this will face further legal challenge—particularly because it is still possible for US intelligence agencies to access EU-derived data [43].

In absence of an adequacy decision or appropriate safe-guards, a transfer to a third country could take place if the data subjects have ‘(…) explicitly consented to the proposed transfer, after having been informed of the possible risks of such transfers for the data subject due to the absence of an adequacy decision and appropriate safeguards’ [44]. One possible concern for im-plementation of DSS in the EU is that patients will be reluctant to consent to the transfer of their data outside the EU. It should be noted that patient consent will not be necessary if the data trans-fer is only for the purposes of providing health care in emergency situations in which the data subject is physically or legally inca-pable of giving consent [45]. The GDPR allows Member States some discretion to introduce further limitations on the transfer of health data to a third country (in the absence of an adequacy de-cision), for example to prevent transfers on the basis of consent [46].

## 3 Conclusions

The clinical application of increasingly sophisticated deci-sion support systems such as Google’s DeepMind, IBM’s Wat-son and Oncoguide, presents a number legal challenges for both DSS-suppliers and healthcare providers. A first set of challenges relate to the professional standard of care and medical liability in case of errors or malfunctioning of systems, either on the part of the healthcare providers using and interpreting the results of the system, or the system itself. Determining a reasonable standard of care when using DSS will not be straightforward. Meeting these challenges therefore, requires preliminary testing and ro-bust translational research. Such research should involve care providers, who need to establish reasonable standards for the use of DSS, and suppliers, who need to ensure they that comply with care standards and meet their obligations under the Euro-pean Regulation on Medical Devices. Regarding liability, it is care providers who are directly liable to patients for failures in their care, but it will not be straightforward to determine whether they have used reasonable skill and care in their consultation and use of DSS. Although DSS suppliers have no direct relationship with patients, they are likely to be subject to liability for errors in the system that result in harm.

A second set of challenges will be meeting legal and eth-ical standards for protection of patient privacy, medical confi-dentiality and data protection. This should not be difficult if an individual’s data are only shared with other professionals for the purpose of providing their care, the data are kept safely and se-curely, not stored or processed for other purposes and do not cross EU-borders. In this case, patient consent will not be strictly necessary [47]. There will be a greater challenge for healthcare providers and DSS-suppliers if patients’ data are used for other purposes (such as research) or if data are processed outside the EU. A major issue will be whether, and if so, under what con-ditions, DSS-suppliers may use the generated data for their own (business) purposes. In all those situations, data controllers are advised to ensure that patients are well informed and are asked for their consent, especially if the use involves commercial pur-poses. If patient data are transferred outside the EU this may be to a country that the European Commission has determined of-fers adequate protection or based on safeguards such as model contractual clauses.
